# A green-fluorogenic, cysteine-reactive dye enables thermal shift-based high-throughput screening with membrane proteins

**DOI:** 10.1016/j.jbc.2026.113571

**Published:** 2026-09-17

**Authors:** Anselm F.L. Schneider, Michelle Keifler, Yung-Ning Chang, Jörg Weiske, Vera Pütter

**Affiliations:** Nuvisan ICB GmbH, Lead Discovery Berlin, Protein Technologies, Berlin, Germany

**Keywords:** biophysics, thermal shift assay, Membrane proteins, binder screen, GPCR, Ion chanel, transporter, Sphingosine-1-phosphate transporter SPNS2, G proteinactivated inward rectifier potassium channel 4, A2AR, beta2AR

## Abstract

Thermal shift assays are useful tools in the biophysical screening of protein targets. They require little assay establishment and no tool compounds, making them particularly useful for proteins with no known natural ligands. Their use in compound-screening is typically limited to soluble proteins, however, as membrane proteins that are solubilized in detergent cannot be routinely used with the fluorogenic dyes that detect hydrophobic surfaces upon protein unfolding. As an alternative, a method has been described that utilizes the frequent occurrence of cysteines within the transmembrane region of membrane proteins in combination with fluorogenic, cysteine-reactive dyes to record melting curves of membrane proteins. Here, we evaluate several novel candidate dyes and find that the dye Naph-EA-Mal can reliably record melting points of membrane proteins at low concentrations and with high tolerance for different buffers or classes of membrane proteins. We apply the thermal shift assay with the new dye to a buffer optimization for the ion channel GIRK4 and to a fragment screen with the solute carrier Spns2. Thereby, we discover a new binder scaffold that may be useful in the development of Spns2 inhibitors in the future.

The discovery of novel therapeutics relies on the ability to identify compounds that interact with biological targets. Classical activity-based high-throughput screening has provided tremendous advances in medicinal chemistry. Nevertheless, it can suffer from limited sensitivity, difficulty developing efficient activity-based assays, and an inability to gain mechanistic insights into the inhibition of compounds. Biophysical screening techniques such as affinity selection-mass spectrometry or surface plasmon resonance (SPR) can directly address some of these limitations and have already proved invaluable in drug screening campaigns (1). Using biophysical screening, the development of an activity-based assay is not necessary (2), and a known natural ligand or tool compound is also not required, making these methods valuable for targets such as orphan receptors (3).

Thermal shift assays (TSAs) make use of ligand-dependent changes in the melting temperature of protein targets (2). We and others have successfully applied TSA in the high-throughput biophysical screening of drug targets, identifying novel inhibitor scaffolds from libraries containing thousands of compounds (4, 5). Aside from screening lead-like compounds, TSAs have also been used to screen fragment libraries, a cost-effective way to probe a large chemical space (6).

TSA is typically performed using environmentally sensitive fluorophores, that show an increase in fluorescence upon binding to the hydrophobic core of unfolding proteins (7, 8). These dyes are generally not applicable to membrane proteins, however, as purified membrane proteins are usually solubilized in detergents, which already provide a hydrophobic environment and trigger the high fluorescence of the environmentally sensitive fluorophores. Membrane proteins still represent the targets of 44% of approved drug substances, (9) however, making the development of biophysical methods for membrane proteins highly attractive.

As an alternative to conventional TSA dyes, an assay using a cysteine-reactive, fluorogenic dye was developed (10): N-[4-(7-diethylamino-4-methyl-3-coumarinyl)phenyl]maleimide (CPM). The assay utilizes the high frequency of cysteines in helix-helix interaction sites, which translates to a high frequency of cysteines in the transmembrane regions of multipass membrane proteins (11). As membrane proteins unfold, these cysteines become solvent exposed, allowing the dye to react, resulting in a fluorescence increase.

Although the “CPM-Assay” is useful to optimize buffer conditions for crystallography (12) or test stabilization with known binders (13, 14), it sees infrequent use in compound screening. This may be due to CPM having an excitation wavelength in the UV range, in which many lead-like compounds absorb light themselves, making assay development more challenging (15). As a result, to our knowledge, there is no published screen of a compound library using the CPM assay.

Here, we compare CPM to three green fluorescent alternatives, including the previously published BODIPY-FL-Cystine (16). We identify a highly fluorogenic dye (Naph-EA-Mal, 2-butyl-6-[2-(2,5-dioxopyrrol-1-yl)ethylamino]benzo[de]isoquinoline-1,3-dione), with a high reaction rate that can reliably record melting curves for representatives of three different types of multispanning membrane proteins. We go on to demonstrate the application of our newly developed protocol in a buffer optimization and in the biophysical screen of a fragment library, identifying a new binder scaffold for an SLC transporter in the process.

## Results

### Development of green-fluorescence-based thermal shift assay with G–protein coupled receptors

Along with the published CPM and BODIPY-FL-Cystine dyes, we identified two green-fluorogenic and cysteine-reactive dyes from the literature: BODIPY 8-P2M (17) and Naph-EA-Mal (18). Like the CPM dye, both alternatives contain maleimide functionalities. The resulting fast thiol labeling may be required to detect thermal unfolding with high sensitivity. To evaluate fluorogenicity of all the dyes, we began by incubating the dyes with the peptidic thiol glutathione in buffer. BODIPY 8-P2M and Naph-EA-Mal had nearly no detectable fluorescence in the absence of thiol but showed a strong increase in green fluorescence after incubation with 0.1 equivalents of thiol (Fig. S1). BODIPY-FL-Cystine in contrast had higher starting fluorescence, which only increased upon addition of at least 1 equivalent of thiol. The CPM dye showed no appreciable green fluorescence with a blue excitation wavelength (at 488 nm).

Next, we attempted to generate melting curves with all fluorophores. For this, we purified the beta-2-adrenergic receptor (β2AR), a well- characterized G–protein coupled receptor, in detergent (Purification in Fig. S2). The protein contains 11 cysteines, of which four are in disulfide bonds and five are unbound and in the transmembrane helices of the protein based on structural data (19). These are the cysteines that would become accessible as the protein unfolds. The protein shows a melting point at 49 °C in nanoDSF (differential scanning fluorimetry using changes in fluorescence from aromatic amino acids), which is increased to 61 °C in the presence of the weak antagonist atenolol (Fig 1). This strong shift in melting temperature is useful in the evaluation of a thermal shift assay. We then performed an initial condition screen with 200 nM and 1 μM protein with two and five equivalents of each fluorophore, monitoring green fluorescence (All conditions shown in Fig. S3). Using a green fluorescence filter, the CPM dye showed a melting curve in the TSA assay, but only at the higher protein concentration and with a low signal/noise ratio. We could not generate any melting curve with the BODIPY-FL-Cystine dye under these conditions, which may be due to the low protein and dye concentrations used (Fig. S3). BODIPY 8-P2M and Naph-EA-Mal however showed a clear melting transition at the expected temperature (based on nanoDSF) and could both be used at the lower protein concentration (Fig. 1).

**Figure 1:**
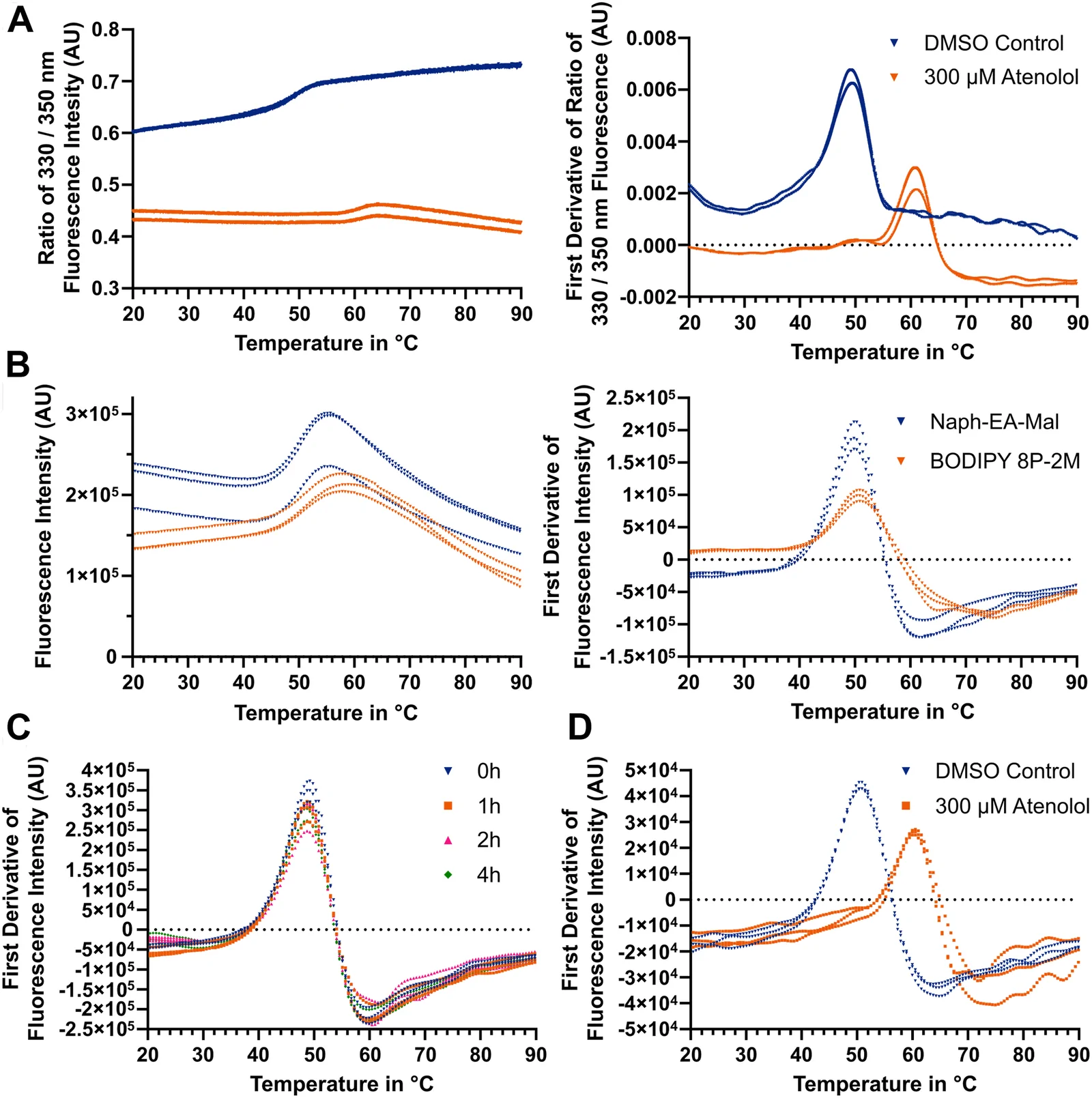
**Evaluation of cysteine-reactive, fluorogenic dyes in generating melting curves.***A*, NanoDSF measurement of detergent-purified β2AR. Shown are the ratio of fluorescence at 330 and 350 nm (*left* graph) and the first derivative of this ratio (*right* graph). *B*, green fluorescence values of the detergent-purified β2AR at 200 nM protein concentration with 1 μM of either the BODIPY 8P-2M or Naph-EA-Mal dyes (*left* graph) and first derivative of the fluorescence intensity from the same experiment (*right* graph). Shown are individual values from three replicates. *C*, replicates of the same 384-well plate were prepared and measured after the indicated incubation time at 4 °C. Shown are the first derivatives of the fluorescence intensity, and individual values from three replicates. *D*, the β2AR was diluted down to 50 nM and mixed with a five-fold molar excess of Naph-EA-Mal, along with 300 μM Atenolol or an equal volume of DMSO. Shown are the first derivatives of the fluorescence intensity, individual values from three replicates. DMSO, dimethyl sulfoxide.

We initially continued working with both the dyes but found that buffer solutions containing the BODIPY 8P-2M-dye would lose color over a few hours (Fig. S4). This may be due to an aggregation-based dye-quenching mechanism, as reported for a structurally related dye (20). In our hands, the dye also produced unreliable results in our protein experiments (data not shown), which is likely due to this instability. The Naph-EA-Mal dye on the other hand was stable in buffer and in the protein solution, and we could record very similar melting curves for β2AR after 0 to 4 h of incubation with the dye 4 °C (Fig. 1), which is highly useful for compound screening setups in which several plates of compounds would be screened over several hours. In addition, we could produce melting curves down to a protein concentration of 50 nM in just 5 μl volume (Fig. 1), corresponding to only ∼13 ng of protein to generate a single melting curve.

To challenge our protocol further, we additionally tested the dye on two different constructs of the adenosine receptor A2A (purification in Fig. S5). The A2AR contains 14 cysteines, of which eight are in disulfide bonds and five are unbound and located within the transmembrane helices (21). Similarly to β2AR, the A2A receptor showed a melting curve under identical conditions, and we could detect a shift in the melting point in the presence of the antagonist ZM-241385 (Fig 2). TSAs for membrane proteins can suffer from not being able to properly record melting curves above a certain temperature (22). We purified a recently published mutant of the A2A receptor that contains the thermostable b562RIL protein inserted into its third intracellular loop (21). This construct has a reported melting point of above 70 °C, which we could reproduce with our assay (Fig. 2). We could also still detect a clear shift in melting temperature in presence of the inhibitor, confirming that the assay is still able to distinguish between apo- and antagonist-bound-states even at very high melting temperatures. To confirm whether the dye labels the proteins covalently as expected, we also performed a labeling experiment and separated the unbound dye from the protein by gel electrophoresis. In-gel fluorescence shows the fluorophore in the protein bands, suggesting that indeed, the protein is labeled covalently (Fig. S6).

**Figure 2:**
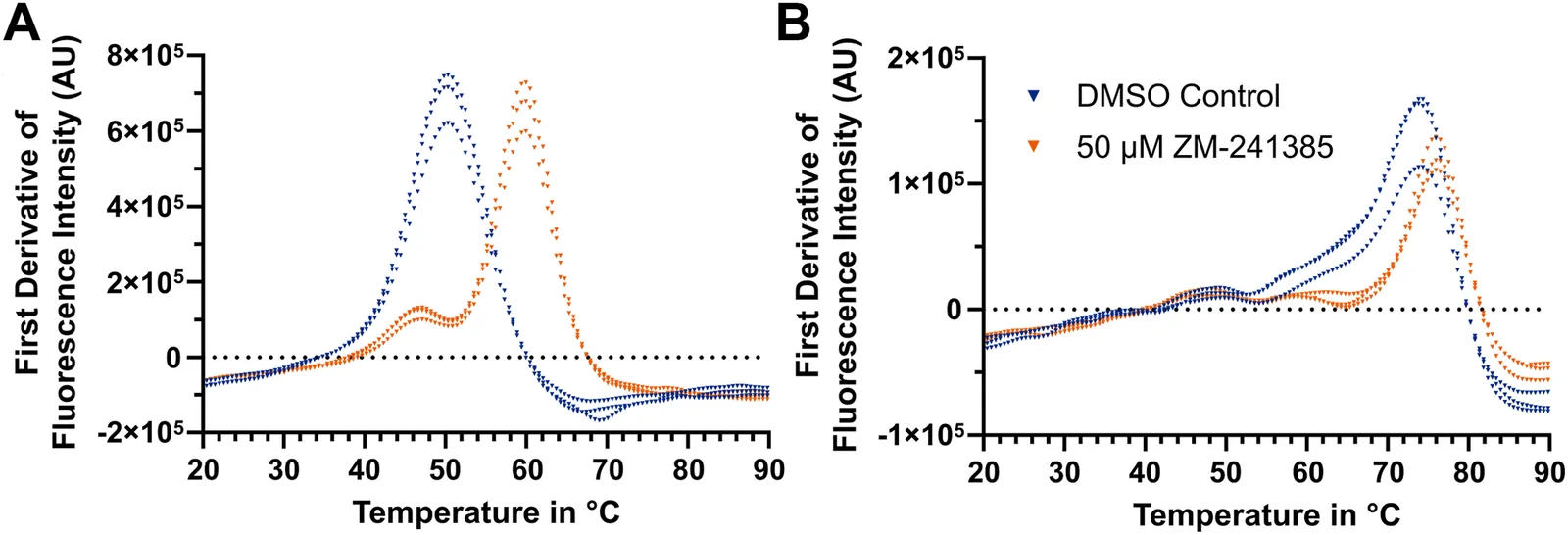
**Application of Naph-EA-Mal fluorophore in thermal shift assay of the adnosine receptor A2A.***A*, melting curves of the detergent-purified A2A receptor in absence and presence of the inhibitor ZM-241385. Shown are the first derivatives of the fluorescence intensity, individual values from three replicates. *B*, melting curves of the detergent-purified, highly stabilized A2A receptor construct “PSB1” in absence and presence of the inhibitor ZM-241385. Shown are the first derivatives of the fluorescence intensity, individual values from three replicates.

### TSA-based buffer additive screening for GIRK4 ion channel

The “G protein–coupled inwardly rectifying potassium channel” (GIRK) family of ion channels are activated through G–protein coupled receptors (23). The GIRK4 channel forms heterotetramers or homotetramers, is primarily found in the adrenal gland and heart, and mutations in its associated gene can lead to hypertension (24). To date, the structure of the homotetramer of GIRK4 has not been experimentally determined. According to the alphafold model, the single GIRK4 subunit contains eight cysteines, of which two should be buried within the transmembrane and extracellular regions.

After purification of the homotetrameric GIRK4 in detergent (purification in Fig. S7), the ion channel had a melting point below 40 °C (Fig 3). We decided to perform a buffer additive screen with the protein to both test the additive tolerance of the TSA assay and identify potentially stabilizing buffer additives. These may increase purified yields of proteins, as well as improve the chances of determining a protein structure, as structure determination by cryo-electron microscopy is reportedly dependent on protein stability (25).

**Figure 3:**
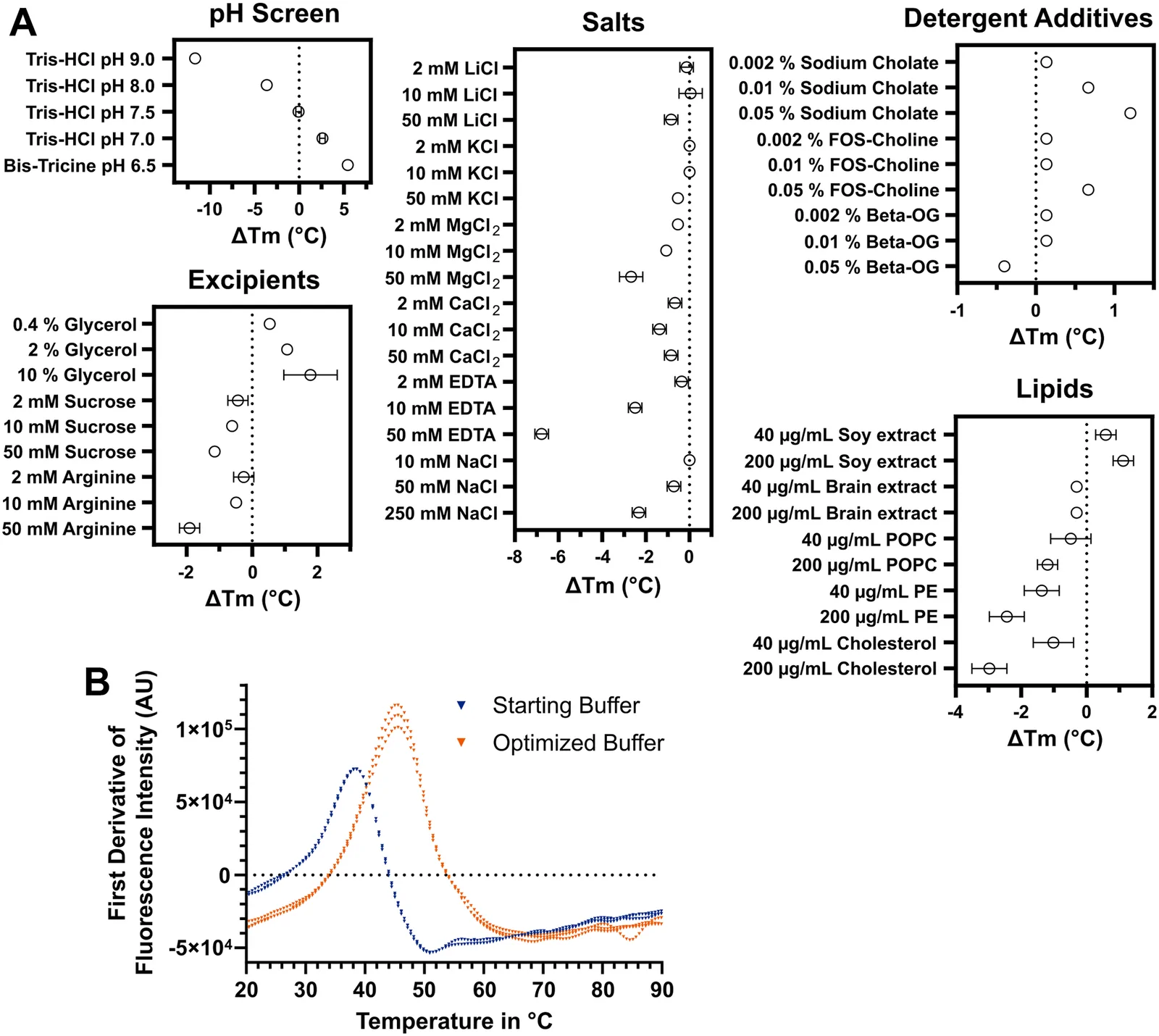
**Results from the TSA-based buffer screen of GIRK4 ion channel.***A*, results from individual buffer modifications. Shown are the average mean change in melting temperature (ΔTm) from three replicates. *B*, comparison of melting curves of the GIRK4 ion channel in the initial starting buffer compared to the optimized buffer. Shown are the first derivatives of the fluorescence intensity, and individual values from three replicates. TSA, thermal shift assay.

We began with a small screen of buffers with different pH values and saw a strong dependence of the melting temperature on the buffer pH (Fig. 3). Using bis-tricine at pH 6.5 showed the highest increase in stability compared to the Tris–HCl pH 7.5 baseline. We tested salt additives and found that they overwhelmingly destabilize the protein, even at low mM concentrations. The common buffer additives arginine and sucrose did not stabilize the protein, but the addition of glycerol to the buffer did. Finally, we also tested several detergents and lipid additives. The detergents fos-choline and sodium cholate, as well as a polar lipid extract from soybean, had a moderately stabilizing effect. Based on these results, we formulated a new protein buffer using bis-tricine at pH 6.5 and including the soy lipid extract and 2% glycerol, which stabilized the protein by around 7 °C compared to the initial starting condition (Fig.3). We envision that this buffer will be useful to improve purification yields and improve odds of structure determination.

### A TSA fragment-screen of the spinster 2 SLC transporter identifies a novel binder scaffold

The solute carrier spinster homolog 2 (Spns2) transports spinghosine-1-phosphate (S1P) from cells into the extracellular space and can thereby regulate circulating S1P levels (26). Inhibiting Spns2 could reduce circulating S1P levels, which may be an attractive way to treat multiple sclerosis indications (27, 28). To speed drug discovery efforts against Spns2, we purified the detergent-solubilized transporter (purification in Fig. S8) (29). The Spns2 protein contains 15 cysteines, of which eight should be unbound cysteines located within transmembrane helices (29).

To identify novel scaffolds that could be used in the development of new inhibitors of Spns2, we performed a TSA screen with a library of chemically diverse fragments. Of the 2000 compounds tested (n = 1), we identified 15 fragments with a ΔTm of over 2.5 °C (Fig 4). One of the fragments, compound 1, lead to an increase in thermostability by 5.1 °C in the initial screen (Fig. 4). After compound-reordering, we could confirm that the fragment causes concentration-dependent thermostabilization of Spns2 (Fig. 4). To validate selective binding, we also performed SPR with immobilized Spns2 and the A2A GPCR as reference. Compound 1 showed selective binding to Spns2 with a 119 μM steady state KD (Figs. 4 and S10). In contrast, we could not record binding in SPR or thermal stabilization of Spns2 with previously published inhibitor SLB1122168 (30). Interestingly, SLB1122168 showed concentration-dependent destabilization of Spns2 in nanoDSF (Fig. S9). In SPR, the SLB1122168 compound showed unselective binding to both membrane proteins (Fig. S10). Both results could be attributed to the high lipophilicity of the compound, which may disturb the detergent-solubilization of the purified protein.

**Figure 4:**
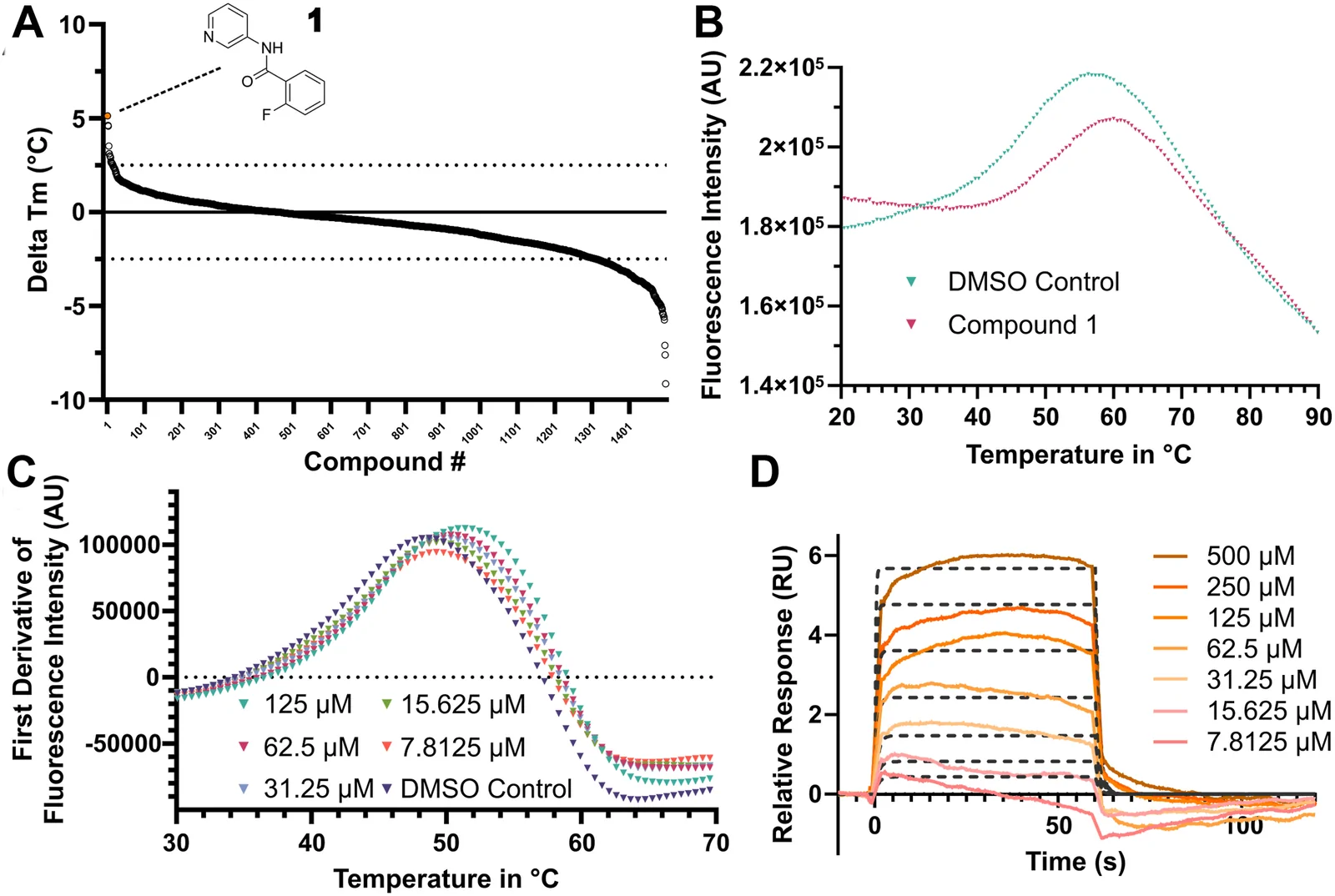
**TSA fragment screen of Spns2.***A*, overview of TSA-based screen of 2000 fragment compounds. The graph shows the change in melting temperature of the tested compounds. Fragments that did not give a clear melting curve are not shown. *B*, raw fluorescence data from the TSA-based fragment screen of a DMSO control and the hit compound 1. *C*, melting curves of spinster 2 with varying concentrations of compound 1. Shown are the mean of three replicates for each compound concentration. *D*, SPR Sensograph of immobilized Spns2 with titration of compound 1. The *dashed gray lines* show the fit used in the calculation of the steady state dissociation constant. DMSO, dimethyl sulfoxide; Spns2, spinster homolog 2; TSA, thermal shift assay.

## Discussion

We have developed a novel thermal-shift assay with the green-fluorogenic, cysteine-reactive dye Naph-EA-Mal. The dye had not previously been used to record melting curves, but is very well suited to this purpose, with its high reaction rate, potent turn-on fluorescence and good aqueous stability. Because of these properties, we were able to record melting curves of several membrane proteins with only low nanogram amounts of protein. As membrane proteins can be highly challenging to produce, miniaturization of the assay is highly valuable for increasing screening capabilities.

The assay is compatible with all major classes of membrane proteins, as we can demonstrate on GPCRs, ion channels and SLC transporters, and with many commonly used detergents such as n-Dodecyl-β-D-maltoside (DDM), lauryl maltose neopentyl glycol (LMNG), cholate, and glyco-diosgenin (GDN). As different membrane proteins will contain different numbers of addressable cysteine residues, assay development may nevertheless be required to identify the ideal ratio of fluorophore to membrane protein. Maleimides are also known to react with primary amines, especially at pH values above 7.5. This may be a limitation for certain screening campaigns, as some proteins may require higher pH. However, the mixture of dye and protein in buffer was stable enough to still reliably record melting curves after several hours of incubation.

We test our established protocol on a challenging target in Spns2, which has few known modulators and a lack of biophysical data in general. From our fragment screen, we identified a novel scaffold that stabilizes the protein and shows selective binding in SPR. The fragment is an attractive starting point and can, in the future, be used to develop new lead compounds.

Screening even larger compound collections is feasible and represents an attractive and efficient way for biophysical screening with membrane protein targets.

## Experimental procedures

### Protein constructs

The Beta-2-adrenergic receptor construct was based on a previously published construct (31) with an N-terminal hemagglutinin signal peptide followed by a Flag-tag and additionally an Avitag on the C terminus for site-directed biotinylation using biotin ligase. The adenosine receptor A2A constructs consist of an N-terminal hemagglutinin signal peptide followed by a Flag-tag and the amino acids 2 to 316 of the WT receptor. The constructs additionally contain a C-terminal Avitag for site-directed biotinylation using biotin ligase. The thermostabilized A2A construct contains the previously reported S91K mutation, and the apocytochrome b562RIL protein inserted between amino acids 208 and 219 of the receptor sequence (21). The GIRK4 protein construct contains the amino acids 47 to 375 of the protein, including N-terminal flag- and Avi-tags and C-terminal 3C-protease-cleavable Enhanced Green Fluorescent Protein (EGFP)- and His10-tags. The Spns2 construct contains the full-length coding sequence of the protein, with C-terminal Avi- and Flag-tags.

### Protein production

Protein expression for the GPCRs, GIRK4, and Spns2 was done in Sf9 insect cells using baculovirus generated by the flashBAC method. Cells were infected with baculovirus at 2 × 10ˆ6 cells/ml and cultured at 27 °C and 80% humidity. Expression was done for 48 h (β2AR, A2A) or 72 h (GIRK4, Spns2). Biotinylated proteins (β2AR, A2A, Spns2) were generated by coinfection with a baculovirus encoding for the biotin ligase BirA and the inclusion of 50 μM biotin in the medium during expression.

Cells were harvested by centrifugation, and membrane proteins were solubilized in Hepes-buffer containing 1% LMNG and 0.1% cholesteryl hemisuccinate (CHS) (β2AR, A2A, and Spns2). The β2AR, A2A and Spns2 proteins were purified *via* Flag M2 affinity chromatography, with gradual reduction of the detergent concentration. After elution with flag peptide, the proteins were further polished by gel filtration on Superdex 200 Increase 10/300 GL (β2AR, A2A) or Superose 6 Increase 10/300 GL (Spns2) columns in a buffer containing 20 mM Hepes pH 7.4, 150 mM NaCl, 0.01% LMNG and 0.001% CHS.

The GIRK4 homotetramer was solubilized in a Hepes-buffer containing 150 mM KCl, 20 mM imidazole, protease inhibitors and 2% DDM. The protein was purified by immobilized metal-affinity chromatography (IMAC) with decreasing concentrations of DDM and 40 mM imidazole. The protein was eluted from the column using 400 mM imidazole and then desalted into a buffer containing no imidazole 20 mM DTT. It was then treated with 3C protease to cleave off the EGFP and his-tags. The protease and EGFP were removed by passing the protein through an immobilized metal-affinity chromatography column. The protein was then further purified by gel filtrationon a Superdex 200 Increase 10/300 column in a Tris-buffer containing 150 mM KCl and 0.05% DDM.

### General nanoDSF protocol

For nanoDSF, proteins were diluted in their respective gel filtration buffers to 0.2 mg/ml concentration. Compounds or an equivalent volume of dimethyl sulfoxide (DMSO) were added to the diluted proteins and the samples were incubated on ice for 30 min. Capillaries were then filled and placed into the Prometheus device (Nanotemper technologies). Melting curves were recorded between 20 °C and 95 °C with a 1.5 °C/min ramp time.

### General TSA assay protocol

All steps were performed on ice up to the TSA measurement, as the proteins have relatively low thermostability. As a starting condition, proteins were generally used at 200 nM concentration with 5 equivalents (1 μM) of fluorophore in their respective gel filtration buffer, unless stated otherwise in the experiment description. Ideal concentrations of protein and fluorophore were determined experimentally for the GIRK4 and Spns2 proteins. Five microliters of the mixture of protein and fluorophore were then pipetted onto a 384-well PCR plate. TSA measurements were recorded on the Quantstudio 7 Q-PCR device, with a 1-min incubation at 15 °C, followed by a temperature ramp of 0.1 °C/s between 15 and 95 °C (the measurement of one plate took approximately 14 min). A blue excitation filter (∼470 nm) was used, with fluorescence readout at 520 nm.

The fragment screen was done with ∼2000 individual fragment compounds prepipetted onto 6384-well plates along with DMSO controls. The final concentration of fragments in the screen was 1 mM.

### Surface plasmon resonance

Binding affinities of compounds were determined using a biacore T200 machine (Cytiva). Biotinylated Spns2 and A2A receptor were immobilized on SA sensor chips to target levels of 1800 to 3000 RU. Compounds were serially diluted in running buffer (20 mM Hepes pH 7.5, 150 mM NaCl, 0.005% LMNG, 0.0005% CHS, and 0.1% bovine serum albumin) from 500 μM down to 7.8 μM. The control compound SLB1122168 was diluted from 5 μM down to 0.078 μM. The diluted compounds were injected over the sensor in running buffer at 16 °C with monitoring of binding traces. Data fitting was performed with a 1:1 interaction model using the biacore insight evaluation software (Cytiva). For KD determination, five replicates of the titration were performed, and a nonlinear curve fit was applied (Fig. S9).

## Data Availability

Data not contained in the supporting information will be made available from the authors upon request.

## Supporting information

This article contains supporting information.

## Conflict of interest

The authors have filed a patent application for this method. All authors are or have been employees of Nuvisan ICB GmbH.
