# Supplementary material for: A green-fluorogenic, cysteine-reactive dye enables thermal shift-based high-throughput screening with membrane proteins
Source: J Biol Chem. 2026 Sep 17;302(11):113571. doi: 10.1016/j.jbc.2026.113571 (PMC13681967; doi:10.1016/j.jbc.2026.113571)
Supplement: Supplementary file 1 [file mmc1.docx]

## A green-fluorogenic, cysteine-reactive dye enables thermal shift-based high-throughput screening with membrane proteins

Anselm F. L. Schneider^1^, Michelle Keifler^1^, Yung-Ning Chang^1^, Jörg Weiske^1^, Vera Pütter^1^

[1] Nuvisan ICB GmbH, Berlin, Germany.

Supporting Information

SI Figure 1. Fluorescence scans of 10 µM cysteine-reactive dyes in buffer, alone or in presence of glutathione or 1 mM TCEP. Dyes were incubated for 1 hour at room temperature, and fluorescence was measured from 495-700 nm with a 485 nm excitation wavelength. From top to bottom: CPM (7-Diethylamino-3-(4'-Maleimidylphenyl)-4-Methylcoumarin), BODIPY FL L-cystine, BODIPY 8-P2M and Naph EA-Mal.

SI Figure 2. SDS-PAGE gel showing the purification steps for the β-2 adrenergic receptor. Gel lanes from left to right: Pageruler prestained protein ladder, total cell lysate, clear cell lysate, affinity chromatography flowthrough, wash steps 1-3, affinity chromatography elution, pooled gel filtration fractions, bovine serum albumin standards 1-3.

 SI Figure 3. Whole data set of melting curves generated with the beta-2 adrenergic receptor (Beta2AR) with all four fluorophores. Shown are the fluorescence intensity, individual values from three replicates. Left graphs include the whole range of the graph, the insets to the right contain a truncated y-axis to show the lower protein concentrations.

 SI Figure 4. Photograph of tubes containing 10 µM of the fluorophores CPM (**1**), BODIPY FL L-cystine (**2**), BODIPY 8-P2M (**3**) and Naph EA-Mal (**4)**, diluted in tris-buffer. The tubes were incubated at room temperature in the dark for the indicated times.

SI Figure 5. Left: SDS-PAGE gel showing the purification steps for the “wildtype” adenosine receptor A2A. Gel lanes from left to right: Pageruler prestained protein ladder, total cell lysate, clear cell lysate, affinity chromatography flowthrough, affinity chromatography elution (5 µl and 1 µl), pooled gel filtration fractions (5 and 1 µl), bovine serum albumin standards 1-4. Right: SDS-PAGE gel showing the purification steps for the “thermostabilized” adenosine receptor A2A containing the bRIL insertion in the intracellular loop 3. Gel lanes from left to right: Pageruler prestained protein ladder, clear cell lysate, affinity chromatography flowthrough, wash steps 1-3, affinity chromatography elution (5 µl), pooled gel filtration fractions (1 and 5 µl), bovine serum albumin standards 1-3.

SI Figure 6. The two GPCRs Beta-2AR (β-2 adrenergic receptor) and A2AR were either left chilled (“intact”) or heated to 95°C for 10 minutes (boiled), then incubated with 2 or 5 equivalents of Naph-EA-Mal, or no dye as control, for 1 hour at room temperature. The samples were mixed with SDS-PAGE sample buffer and run on SDS-PAGE. The gel was first imaged for in-gel fluorescence with broad UV excitation (right side), then stained using coomassie stain (left side). The fluorescence coincides with the protein bands, suggesting that the labelling persists under SDS-PAGE conditions and is therefore likely covalent.

SI Figure 7. SDS-PAGE gel showing the purification steps for the GIRK4 homotetramer. Gel lanes from left to right: Total cell lysate, clear cell lysate, IMAC flowthrough, IMAC wash fraction, IMAC elution, desalting elution, concentrated desalting elution, input into reverse IMAC/gel filtration, pooled gel filtration fractions (0.7 and 10 µl), pageruler prestained protein ladder.

SI Figure 8. SDS-PAGE gel showing the purification steps for the Spinster 2 protein. Gel lanes from left to right: Pageruler prestained protein ladder, total cell lysate, clear cell lysate, affinity chromatography flowthrough, affinity chromatography wash fraction, affinity chromatography elution, concentrated affinity chromatography elution (input for gel filtration), pooled gel filtration fractions (10 and 5 µl), bovine serum albumin standards 1-3.

SI Figure 9. NanoDSF measurement of detergent-purified Spns2 in presence of various concentrations of SLF1081851 or equivalent volume of DMSO. Shown are individual values of three replicates of the first derivative of the fluorescence at 350 nanometers.

SI Figure 10. A: SPR Sensograph of immobilized Spns2 protein with titration of compound 1. The dashed grey lines show the fit used in the calculation of the steady state dissociation constant. B: SPR Sensograph of immobilized A2A receptor with titration of compound 1 as control. C: SPR saturation curve from five replicate SPR experiments of a KD titration experiment of compound 1 on Spns2. Shown are the mean and standard deviation, and a non-linear curve fit. Fit details are shown in SI Table 1. D: SPR saturation curve from five replicate SPR experiments of a KD titration experiment of compound 1 on A2AR. Shown are the mean and standard deviation. E: SPR Sensograph of immobilized Spns2 protein with titration of compound SLB1122168 as control. F: SPR Sensograph of immobilized A2A receptor with titration of compound SLB1122168 as control.

SI Table 1: Fit data from the non-linear curve fit (see SI Fig. 10F) for KD determination of compound 1 binding to SPNS2 in five replicate SPR experiments.

| **Best-fit values** |  |
| --- | --- |
| Bmax | 7,069 RU |
| Kd | 118,6 µM |
| **95% CI (profile likelihood)** |  |
| Bmax | 6,035 to 8,451 RU |
| Kd | 79,32 to 180,4 µM |
| **Goodness of Fit** |  |
| Degrees of Freedom | 35 |
| R squared | 0,9052 |
| Sum of Squares | 13,22 |
| Sy.x | 0,6146 |
